# Interprofessional education between medical students and nurse practitioner students in a Global Health course

**DOI:** 10.1186/s12909-018-1307-y

**Published:** 2018-08-17

**Authors:** James S. Leathers, Heather Davidson, Neerav Desai

**Affiliations:** 1 Vanderbilt University School of Medicine, 2209 Garland Ave., Nashville, TN 37232 USA; 2 Vanderbilt University Medical Center, 1211 Medical Center Dr, Nashville, TN 37232 USA; 3 Vanderbilt Health One Hundred Oaks, 719 Thompson Lane Suite 36300, Nashville, TN 37204 USA

**Keywords:** Interprofessional education, IPE, Medical education, Global health

## Abstract

**Background:**

Few global health experiences include intentionally-directed interprofessional training. We aim to prospectively evaluate the impact of a global health elective in facilitating interprofessional education (IPE) and promoting cultural sensitivity.

**Methods:**

We included in our study, medical and nursing students who participated in the 2015 and 2016 cohorts of the Nicaragua Global Health course. The course consisted of a 12-week curriculum, and included an in-country immersion where students were organized into small-groups that participated in a variety of interprofessional activities. Students filled out pre- and post-course surveys. We performed quantitative analysis on numeric data and qualitative analysis on open-ended questions.

**Results:**

Of 39 total students enrolled in the course, 26 (18 medical and 8 nursing students) participated in the study and filled out the pre- and post-course surveys. Mean competency scores increased for all questions between pre- and post-course surveys, and of these, 5 of 7 reached statistical significance. Qualitative themes identified included: 1) the importance of understanding other team member’s roles and relative strengths; 2) the value provided by the breaking down of traditional power dynamics between clinicians.

**Conclusions:**

Global health experiences represent a unique and under-utilized opportunity for facilitating IPE.

## Background

Global health education can be an excellent adjunct to the development of health professionals because it challenges students to view complex problems from multiple perspectives [1]. In an effort to improve healthcare team communication, efficiency and cohesiveness, interprofessional education (IPE) has emerged as a modern tool in healthcare education [2]. As the curricular goals of global health align well with those of IPE, experiences that combine both teaching modalities may have complementary benefits. By being exposed to different professional norms that exist in foreign cultural settings, professional students may transfer the cultural humility they gain while immersed in-country, to their future work as part of an interprofessional team [3–5].

Although there remains no universally agreed upon best methodology for teaching interprofessional skills, a recently published review of 28 educational programs found nine major education strategies currently available for the implementation of IPE, with community and rural clinical rotations being among the most commonly used [6]. Studies of interprofessional clinical rotations in community and rural settings show that these experiences increase students’ confidence in caring for underserved populations and affect long-term changes in practice behavior through the modification of attitudes, values and competencies [6]. While many clinical programs have been established in the US and internationally, few clinical rotations include global health experiences where the students spend time abroad participating in intentionally-directed interprofessional training. This is an unfortunate reality, as previous studies have found that international clinical rotations promote sensitivity to cost issues, decrease reliance on technology and laboratory diagnostics, increase appreciation for cross-cultural communication and increase the proportion of students who wish to practice in low-resource communities [7]. In light of these benefits, a large opportunity exists for students in which to acquire these same skills and values, meanwhile learning how to work side-by-side with other healthcare professionals.

The Nicaragua Global Health Course is an interprofessional elective that has been available since 2012 and represents a unique opportunity for such learning. Previous course evaluations have indicated positive student experiences. However, our course’s impact on student perceptions and beliefs, as well as understanding of global contexts and interprofessional roles and responsibilities, remains unknown. In this study, we aim to prospectively evaluate the effect of an interprofessional global health elective in facilitating IPE and promoting cultural sensitivity.

## Methods

### Course design

**Fig. 1:**
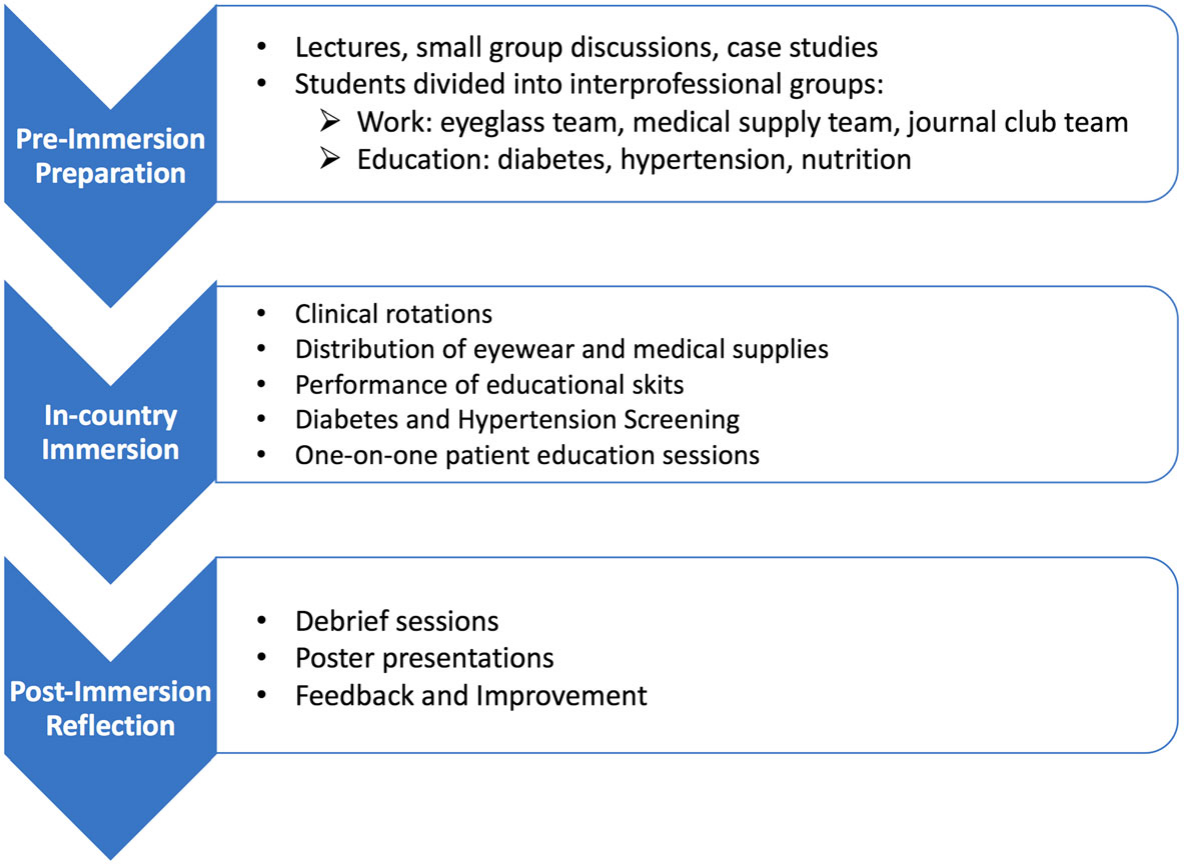
The three phases of the Nicaragua global health course

The Nicaragua Global Health Course was implemented at Vanderbilt University in collaboration with The Vanderbilt Institute of Global Health, The Center for Latin American Studies, The Vanderbilt University School of Medicine, and the Vanderbilt University School of Nursing. It consisted of a 12-week curriculum conceptually divided into three phases: 1) pre-immersion preparation, 2) in-country immersion in Nicaragua, and 3) post-immersion reflection (Fig. 1). [Fig. 1 near here]. Course faculty from both the Vanderbilt School of Medicine and the Vanderbilt School of Nursing, were present at every phase of the course.

#### Pre-immersion preparation

This portion of the course consisted of ten weeks of didactic lectures and small group discussions that were designed to introduce students to the concepts of interprofessional collaboration. Culturally relevant case studies were used to draw out potential areas of conflict and discuss the differences and similarities of medical and nursing roles. During the pre-immersion preparation phase, students were divided into three work teams, according to student’s interests. The first team was assigned the task of planning and orchestrating a journal club for US and Nicaraguan students/providers. The second group was responsible for the collection of donated prescription and reading glasses in the US, and the distribution of these glasses to Nicaraguan patients with matching prescriptions. The third group was responsible for collecting medications, surgical supplies, and other medical devices in the US, to take to Nicaragua. In addition to the work groups, students were also divided into three educational groups that were tasked with designing patient education materials for diabetes, cardiovascular disease, and nutritional health. These topics were chosen at the behest of our Nicaraguan medical colleagues who mentioned that chronic disease education was sorely lacking due to insufficient clinical time. Students met once a week in the pre-immersion preparation to design culturally-appropriate, educational skits in Spanish, that focused on health literacy within these topics. Students also designed educational handouts that were given to patients in clinic. Course faculty gave lectures during didactic sessions, facilitated small group discussions, connected students to organizations responsible for donating eyeglasses and medical supplies, and approved final versions of patient educational skits and handouts.

#### In-country immersion in Nicaragua

The immersion phase consisted of a seven-day, in-country experience, where students were engaged in various clinical settings. Two days were spent at the Nicaraguan Eye Hospital in Managua, Nicaragua. In the mornings, students rotated either through the clinic, the operating room or the emergency department, and in the afternoons, students distributed medications and eye glasses to patients. Three days were spent at a primary care clinic, in Granada, Nicaragua. In this clinic, medical students and nursing students worked side-by-side to treat patients with local and US providers, and performed educational skits for patients in the waiting room. After patients finished their visit, they were given the opportunity to sit with students in one-on-one patient education sessions. Finally, students coordinated hypertension and diabetes screenings by offering blood pressure and blood glucose measurements, respectively. Course faculty coordinated with local Nicaraguan colleagues to arrange specific dates for students to perform clinical rotations.

#### Post-immersion reflection

After returning from Nicaragua, a debrief meeting was held where students were asked to share their experiences and discuss what they learned. Students also met to prepare a poster presentation on patient education and teaching, that was presented to the Vanderbilt medical center community. Feedback from this session provided information that was used to improve the course for each successive year.

### Student participants and course surveys

We included in our study, medical students (M.D. track, 4-year program) and nurse practitioner students (Masters in Science of Nursing [MSN] track, 2-year program) from the 2015 and 2016 cohorts of the course. All students were in the first year of their respective degree programs. As participation in the study was voluntary, all students underwent signed informed consent prior to the commencement of the course. Students that agreed to participate were sent an electronic survey at both the beginning and the completion of the course. The course survey was designed by course faculty to reflect the learning objectives of the course. Study data were collected and managed using REDCap electronic data capture tools hosted at Vanderbilt University [8]. REDCap (Research Electronic Data Capture) is a secure, web-based application designed to support data capture for research studies, providing: 1) an intuitive interface for validated data entry; 2) audit trails for tracking data manipulation and export procedures; 3) automated export procedures for seamless data downloads to common statistical packages; and 4) procedures for importing data from external sources.

The pre- and post-course survey included binary questions (i.e. yes or no), questions in which students were asked to respond to a single statement on a 100-point slider scale, as well as open-ended free-response questions. The course directors created the survey items to directly correspond to the goals and objectives of the course and to align with IPE competencies. Some questions were specific to either medical or nursing students. Due to small class sizes, age and gender information was not recorded in order to preserve confidentiality of participants. This study received approval from our institution’s Institutional Review Board.

### Quantitative analysis

Summary statistics were performed and continuous variables were expressed as means (range), and categorical variables were expressed as proportions (%). Due to relatively small sample sizes, the Fischer’s Exact test was used to compare categorical variables. Due to the non-parametric distribution of certain variables, the Mann-Whitney U test was used to compare continuous variables. A *p*-value ≤ .05 was taken as the criterion for statistical significance. Statistical analysis was performed using STATA v14.2 (Statacorp, College Station, TX).

### Qualitative analysis

We analyzed the open-ended questions in both the pre- and post-course surveys and identified unifying themes between student responses. Quotations that supported the identified themes were excerpted.

## Results

### Quantitative results

Of 39 total possible participants in the two cohorts, 26 (66.6%) agreed to partake in our study and filled out pre-course and post-course questionnaires. Our study includes 18 medical students and 8 nursing students from the Vanderbilt University School of Medicine and the Vanderbilt University School of Nursing, respectively. While most students (73%) had previously participated in an international learning experience prior to the course, just 35% of students had participated in an interprofessional learning experience. However, 63% of nursing students had previous IPE experience, compared to only 22% of medical students. Pre- and post-survey questions that related to IPE and team-building were reviewed for significant changes. Mean competency scores increased for all questions between pre- and post-course surveys, and of these, 5 of 7 reached statistical significance (Table 1). [Table 1 near here].

**Table 1:** Quantitative Data Comparing Pre-Course and Post-Course Competencies

	Pre-Course	Post-Course	P
Q1. Effectively communicate your own profession’s roles and scope of practice to other health professionals	66.0 (28–100)	81.5 (63–100)	< 0.001
Q2. Explain the roles and scopes of practice of both a Registered Nurse & Advanced Practice Nurse colleague in a variety of care settings	28.2 (0–61)^1^	65.3 (34–88)^1^	< 0.001^1^
Q3. Explain the roles and scopes of practice of a physician colleague in a variety of care settings	64.7 (26–85)^2^	78.8 (50–97)^2^	0.061^2^
Q4. Listen actively to people expressing different perspectives than your own	76.5 (25–100)	85.5 (61–100)	0.128
Q5. Encourage ideas and opinions of other team members during group discussions or debates	67.5 (22–100)	80.5 (52–100)	0.007
Q6. Engage other health professionals in shared patient-centered problem solving	55.6 (30–77)	75 (50–100)	< 0.001
Q7. Describe the process of team development and practices of effective teams	60.9 (23–100)	77.4 (33–100)	< 0.001

^1^ Medical students only (*n* = 18). ^2^Nursing students only (*n* = 8). Parameters expressed as mean (range). Comparisons between groups were made using the Mann-Whitney U test

### Qualitative results

We aimed to assess student’s perspectives and attitudes towards IPE activities after completion of the global health course. Two major themes related to IPE were identified.

The first theme that emerged was the importance of understanding other team member’s roles and relative strengths. When asked about common challenges to providing interprofessional care, one student answered, “different health professions are often only educated on their own roles and they do not understand the roles and abilities of the other members of the team.” Following the course, a student asked to reflect on the different strengths between medical and nursing students, wrote, “nursing students have more practical knowledge in terms of managing the needs of patients while I think that medical students have more foundational understanding of body systems and diseases. I think that we have a lot to learn from each other.” A second student emphasized how the respective training of physicians and nurses gives them a unique perspective of patient care: “Medical students were effective at tackling individual diseases, their pathophysiology and treatment. NPs were better at seeing the bigger picture and had a good grasp of the intangibles of caring for patients.” There appeared to be a general consensus that although nursing and medical students have different training, these differences are advantageous and help complement each other.

The second theme identified was the value provided by the breaking down of traditional power dynamics between physicians and nurses. In reflecting about the roles played by medical and nursing students, one student wrote, “sometimes it is difficult to navigate the hierarchy between physicians and nurses. This was not a problem with our group.” Meanwhile other students wrote, “there actually didn’t seem to be any distinction between us when we were working at clinics” and “the roles were very intermixed while in Nicaragua. There was no true separation between the roles of medical students and nursing students. Rather, we functioned as a cohesive team and completed tasks as a unit.” One medical student, when asked about how this experience would influence their future interactions with their nursing colleagues, they wrote that they would, “always respect their role and communicate as equals.” Lastly, one student eloquently summarized the importance of healthy power dynamics within the medical team: “If we reinforce hierarchies within medicine, we will inevitably project them onto our patients, making them feel like ‘lesser’ team members. By affirming and welcoming the perspectives of interprofessional colleagues we affirm the fact that healthcare is not about us as individuals or our egos, it is about the patients.”

## Discussion

Due to the mounting complexity of patient care and the increasingly specialized nature of individual roles within clinical teams, there exists an acute need to develop educational curricula tailored to the cultivation of interprofessional learning competencies. In this study, we assert that the three phases of our global health course provide a unique environment that is conducive to accomplishing this end. Both the quantitative and qualitative information collected pre- and post-course provide us with valuable insights about the courses’ impact on both medical and nursing students.

Participants self-reported answers demonstrated improvement between pre- and post-course surveys, for all seven of the questions asked on a 100-point scale, however, two of these questions did not reach statistical significance (Q3 and Q4). The failure of Q3 to reach statistical significance is best explained by a small sample size (*n* = 9), as this question was directed only at the nursing students in the study cohort, whom made up a minority of participants. Of note, nursing students were more confident in being able to describe their medical colleague’s roles, both before (mean pre-score: 28.2 vs. 64.7, medical vs. nursing students, respectively) and after the course (mean post-score: 65.3 vs. 78.2). However, medical students had a larger absolute improvement in this competency (difference in pre- and post- score mean: 37.1 vs. 13.5, medical vs. nursing students, respectively). These results suggest that while both student types improved, medical students experienced more benefit from the course with regards to this competency. While medical students and RN-track nursing students often have the opportunity to work together during professional school, medical students rarely have the opportunity to work with nurse practitioner students. This lack of interaction can help explain the low pre-course ability for medical students to describe the role of their nurse practitioner colleagues. During classroom conversations about professional roles, course directors observed that understanding the differences between the two types of nursing training was a significant point of discussion. The dramatic improvement of this competency within medical students highlights the utility of our course as a means of teaching IPE. Furthermore, since the majority of nursing students had already had an IPE experience (63%), it is likely that they had been familiarized with the roles of physicians through their previous curricula. While the difference between pre- and post-course responses to Question 4 also failed to reach statistical significance, it’s worth nothing that Q4 had the highest pre-course value. This indicates that the relatively low margin of improvement in this domain was likely due to a high baseline level of competency among students. Nonetheless, asking students to self-assess their listening skills can also be problematic given that people tend to overestimate their ability. This competency would be better measured by a trained observer.

The first qualitative theme identified in our paper was the importance of understanding other team member’s roles and perspectives on health care delivery. There are few times during traditional medical or nursing training that students have the dedicated space to learn from each other and experience each other’s reflection about their own scopes of practice. When medical and nursing students work side-by-side to accomplish a protocol in the normal clinical setting, they can be too focused on accomplishing their own work to step back and discuss the philosophy of care and foundational knowledge driving their behavior. The beauty of the immersive experience in global health courses, is that it removes people from their daily, normal work flow and allows for more organic discussion and connection to take place. The insights the students provided in their open-ended, qualitative comments about how they better understand the purpose and goals-of-care of their respective clinical counterparts, is testament to this deeper learning.

The breaking down of traditional power dynamics between physicians and nurses was the second qualitative theme identified from open-ended survey questions. From the moment students arrived in-country, they had to apply principles of cultural competency first-hand. A recent review published by Hean et al. found that several studies on IPE courses identified improvement in cultural competence as a necessary outcome [9–12]. While these IPE courses demonstrated benefit in eliciting changes to participant perspectives, none of them included an immersive experience that forced the students to interact with a previously unfamiliar environment. In a global health course, students are placed into a new setting in which many are unaccustomed to the local language, culture, healthcare system etc. The summation of all these different obstacles creates an environment that is not just conducive toward learning interprofessional collaboration, but directly reliant upon it for success. In this immersion, students are “forced” by necessity to work together in such a way that traditional roles and hierarchy (i.e. physician’s overseeing nurses) are cast aside in favor of a system of mutual understanding that prioritizes the end goal (i.e. efficiently distributing eye glasses to hundreds of waiting patients), rather than the means (i.e. assigned physician leaders). By breaking down these historically-fixed roles, the interprofessional relationship begins to take on more of peer-to-peer dynamic rather than superior-to-subordinate dynamic. This allows for open channels of communication and gravitation toward more natural leadership roles (i.e. students with strong Spanish skills become liaisons between group and hospital staff). The long-term benefit of having clinicians with cultural competence extends past the direct relationship between patient and provider. In addition to helping providers connect with patients, cultural competence portends a certain open-mindedness that supports enhanced communication between healthcare providers [13]. In an increasingly globalized world, learning how to adapt and interact with other cultures will assist providers in working with foreign-born colleagues and treating foreign-born patients [14]. In fact, longitudinal studies found that immersive global health courses were associated with an increased preference to work with underserved patient populations, which would address a severe need in the US [15–17]. Finally, providers with this competency will invariably create safer clinical work environments by endorsing respectful two-way communication between team leaders and other team members [18, 19].

Our findings must be interpreted in the context of our study’s limitations. Due to the logistical and financial difficulties of coordinating a large international elective, our course has been limited to 10 students per year. As such, the analysis of quantitative outcomes is limited by low statistical power. Our elective has been more popular among medical students, so we did not have an ideal 1:1 ratio of medical to nursing students. This may have limited the number of interactions that a student could have with another student of a different professional track. Lastly, since the study survey was optional, there exists a potential for a response bias, as students who had a positive experience with the course may be more likely to complete the post-course survey.

The nature of providing clinical and education services in a lower income country such as Nicaragua presents a unique opportunity for IPE collaboration. The collaboration between students is greatly enhanced when common goals of cultural competence, patient education, and problem-solving are emphasized. The immersion phase provides a real-world environment for students to apply the principles of interprofessional collaboration taught during the preparation phase. The course directors observed that by the end of the immersive phase, the teams are working efficiently and smoothly as if no interprofessional barriers existed between them. While Nicaragua does not have nurse practitioners in the traditional sense, course directors frequently witnessed medical students explaining to Nicaraguan physicians the roles and benefits of nurse practitioners in both rural and hospital settings. The ability of students to articulate the role of their clinical counterparts to patients, suggests not only that students understand each other’s professional-tracks, but more importantly, that they value them.

## Conclusions

We conclude from this study that the global health setting presents a unique opportunity for learning IPE competencies. Educators seeking to prepare similar programs aimed at teaching the principles of IPE can use our global health course as a model. Future global health courses and endeavors at our institution will take special note of meeting the competency of interprofessional collaboration and show intent with design and implementation.

## Abbreviation

IPE: Interprofessional education
